# Growth of Ambulatory Virtual Visits and Differential Use by Patient Sociodemographics at One Urban Academic Medical Center During the COVID-19 Pandemic: Retrospective Analysis

**DOI:** 10.2196/24544

**Published:** 2020-12-04

**Authors:** Sarah F Gilson, Craig A Umscheid, Neda Laiteerapong, Graeme Ossey, Kenneth J Nunes, Sachin D Shah

**Affiliations:** 1 Department of Medicine University of Chicago Chicago, IL United States; 2 Center for Healthcare Delivery Science and Innovation University of Chicago Medicine Chicago, IL United States; 3 Digital Health University of Chicago Medicine Chicago, IL United States; 4 Department of Obstetrics & Gynecology University of Chicago Chicago, IL United States; 5 Department of Pediatrics University of Chicago Chicago, IL United States

**Keywords:** telemedicine, telehealth, video visit, telephone visit, virtual visit, COVID-19, age, sex, race, insurance, demographic, retrospective

## Abstract

**Background:**

Despite widespread interest in the use of virtual (ie, telephone and video) visits for ambulatory patient care during the COVID-19 pandemic, studies examining their adoption during the pandemic by race, sex, age, or insurance are lacking. Moreover, there have been limited evaluations to date of the impact of these sociodemographic factors on the use of telephone versus video visits. Such assessments are crucial to identify, understand, and address differences in care delivery across patient populations, particularly those that could affect access to or quality of care.

**Objective:**

The aim of this study was to examine changes in ambulatory visit volume and type (ie, in-person vs virtual and telephone vs video visits) by patient sociodemographics during the COVID-19 pandemic at one urban academic medical center.

**Methods:**

We compared volumes and patient sociodemographics (age, sex, race, insurance) for visits during the first 11 weeks following the COVID-19 national emergency declaration (March 15 to May 31, 2020) to visits in the corresponding weeks in 2019. Additionally, for visits during the COVID-19 study period, we examined differences in visit type (ie, in-person versus virtual, and telephone versus video visits) by sociodemographics using multivariate logistic regression.

**Results:**

Total visit volumes in the COVID-19 study period comprised 51.4% of the corresponding weeks in 2019 (n=80,081 vs n=155,884 visits). Although patient sociodemographics between the COVID-19 study period in 2020 and the corresponding weeks in 2019 were similar, 60.5% (n=48,475) of the visits were virtual, compared to 0% in 2019. Of the virtual visits, 61.2% (n=29,661) were video based, and 38.8% (n=18,814) were telephone based. In the COVID-19 study period, virtual (vs in-person) visits were more likely among patients with race categorized as other (vs White) and patients with Medicare (vs commercial) insurance and less likely for men, patients aged 0-17 years, 65-74 years, or ≥75 years (compared to patients aged 18-45 years), and patients with Medicaid insurance or insurance categorized as other. Among virtual visits, compared to telephone visits, video visits were more likely to be adopted by patients aged 0-17 years (vs 18-45 years), but less likely for all other age groups, men, Black (vs White) patients, and patients with Medicare or Medicaid (vs commercial) insurance.

**Conclusions:**

Virtual visits comprised the majority of ambulatory visits during the COVID-19 study period, of which a majority were by video. Sociodemographic differences existed in the use of virtual versus in-person and video versus telephone visits. To ensure equitable care delivery, we present five policy recommendations to inform the further development of virtual visit programs and their reimbursement.

## Introduction

The COVID-19 pandemic has significantly altered the landscape of health care delivery. One of the major changes resulting from the pandemic has been the rapid adoption of virtual (ie, telephone and video) visits and other telemedicine programs that facilitate health care services via health care information technologies to accommodate necessary reductions in in-person care [1,2]. A major driver for this adoption was the Centers for Medicare & Medicaid Services (CMS) expansion of virtual visit reimbursement on March 17, 2020, under the 1135 waiver authority. This allowed for Medicare reimbursement of multiple visit types performed virtually, including outpatient clinic visits, retroactively starting March 6, 2020, and continuing for the duration of the public health emergency [3]. This shift to reimburse virtual visits helped clinicians continue caring for patients despite widespread shelter-in-place orders and may represent the beginning of a new era for ambulatory medicine.

Unfortunately, access to virtual visits may not be equitable in the United States. Differential access to the internet and devices and differences in health literacy may leave patients without the ability to attend video visits. Thus, those patients may only be able to participate in telephone visits if they are unable to attend in-person visits. Surveys by the Pew Research Center in 2019 found lower rates of internet usage and smartphone ownership among people ages ≥65 years compared to younger adults [4,5]. When examining access to internet and internet technology by race, Black adults had lower rates of access to the internet and lower rates of desktop or laptop computer ownership than White adults [4,6]. A recent study of Medicare beneficiaries found that digital access was lowest among patients who were ≥85 years, Black, or received Medicaid [7]. Additionally, adults who are older, men, and Black have been shown to have lower health literacy levels than those who are younger, women, and White; and low health literacy is associated with a greater likelihood of needing help performing online tasks [8-10]. These disparities in access to the internet and devices and lower health literacy levels may lead to corresponding disparities in health care delivery and quality, particularly if the quality of health care visits and visit satisfaction are greater with video visits compared to telephone visits [11-13]. Furthermore, patients who opted out of virtual visits entirely and continued to attend in-person visits during the pandemic may have increased their risk of exposure to COVID-19 or experienced decreased appointment availability due to the decrease in in-person capacity required to maintain COVID-19 social distancing. Thus, though virtual visits have been considered an integral part of delivery of health care during the pandemic, access to those visits (especially video visits) may have been affected by underlying differences in access to technology and health literacy.

There is already existing evidence that other recent innovations in health care technology may exacerbate differences in health care access. For example, patient portal use, which has the potential to improve the quality and efficiency of health care delivery, differs with respect to race, insurance, and neighborhood broadband internet access [14]. One study found that patient portal use was lower among Black (vs White) patients; Medicare, Medicaid, and uninsured (vs commercially insured) patients; and patients with decreased neighborhood broadband internet access [14]. Other studies using data prior to the COVID-19 pandemic have additionally suggested that telemedicine and patient-facing health information technology utilization is lower among men, patients over 65 years, non-White patients, patients without commercial insurance, and patients living in neighborhoods with low internet access; this lack of internet access and technology proficiency continues to impede wider adoption of health information technology among racial minorities and those without commercial insurance [15-18]. Given prior research on the benefits of telemedicine interventions on clinical outcomes, such as improvement in glycemic control in medically underserved patients with diabetes, these disparities in the use of and access to digital health may directly translate into disparities in health care quality [19].

Despite widespread interest in the use of virtual visits for ambulatory patient care during the COVID-19 pandemic, few studies to date have evaluated the adoption of ambulatory virtual visits during the pandemic by age, race, sex, or insurance [20]. The studies that have been published recently show that patients using virtual visits during the pandemic were more likely to be younger adults as compared to older adults, female, non-White, and not commercially-insured [2,21-23]. This may be due in part to the lack of patient readiness for virtual visits, which one study found was more prevalent in patients who were older, male, or Black, and affected video visits more than telephone visits [24]. However, most of the studies published on data from the pandemic did not evaluate the impact of these sociodemographic factors on the use of telephone versus video virtual visits. Such assessments are crucial to identify, understand, and address differences in care delivery across patient populations, and inform policy decisions, particularly those like reimbursement rules, which could affect access to or quality of care.

In this study, we aimed to (1) assess changes in visit volume, type, and patient sociodemographics from the start of the COVID-19 national emergency to the end of May 2020, compared to the same weeks in 2019; and (2) elucidate differences in the use of ambulatory virtual visits (as compared to in-person visits) and, for those using virtual visits, the use of video visits (compared to telephone visits) by age, sex, race, and insurance. We hypothesize that (1) total visit volumes decreased and virtual visits increased during the COVID-19 pandemic, while patient sociodemographics remained similar between the two time periods; and (2) patients who utilized in-person visits during the COVID-19 study period were more likely to be younger than patients who utilized virtual visits, and of those using virtual visits, patients utilizing video visits were more likely to be younger, White, and have commercial insurance than patients utilizing telephone visits [2,21-23].

## Methods

### Setting

The University of Chicago Medical Center (UCMC) is the flagship institution of University of Chicago Medicine, and includes 5 multispecialty faculty ambulatory practice sites in Chicago, IL, and the surrounding area, with over 600,000 encounters per year. UCMC began offering virtual visits in March 2020 in response to the widespread shelter-in-place orders at the city, state, and regional level due to the COVID-19 pandemic. Telephone visits began during the week of March 15, 2020. Video visits began with a pilot program in the hematology/oncology, pediatrics, psychiatry, gastroenterology, and obstetrics/gynecology practices on March 26, 2020, followed by a broad roll-out to all ambulatory faculty clinics on April 6, 2020. All practices used a HIPAA (Health Insurance Portability and Accountability Act)-compliant Zoom platform to enable video visits, which was not integrated into the institution’s electronic health record system (Epic) during the evaluated time period.

Immediately after the City of Chicago and State of Illinois shelter-in-place orders were enacted, patients with previously scheduled in-person office visits were contacted and given the option to either reschedule or convert their appointment to a virtual visit. If a patient agreed to a virtual visit, a video visit was encouraged. Patients scheduled for video visits were sent the following through the patient portal or email: a Zoom link for the video visit; a brief prevideo visit checklist followed by more detailed instructions describing the technical requirements to participate in the video visit; and a link to a video highlighting methods to best prepare for the video visit and a demonstration of what to expect. If the patient was unable or unwilling to participate in a video visit, a telephone visit was scheduled, and they were told to expect a call from their provider at the scheduled appointment time. Patients reaching out to schedule new virtual visits were also preferentially offered video visits but were given the opportunity to schedule a telephone visit as well in accordance with their preferences. The availability of virtual visits was marketed widely to our patient population through our patient portal, marketing emails, and our health system’s internet home page. Beginning on May 1, 2020, patients were given the option to begin self-scheduling video visits (but not telephone visits) through the patient portal.

### Study Population and Measures

All adult and pediatric outpatient clinic visits occurring in UCMC faculty practice locations from March 15 to May 31, 2019, and March 15 to May 31, 2020, were included. The type of outpatient clinic visit was classified as in-person or virtual, and virtual visits were further classified as telephone or video, based on the scheduled visit type for all completed visits. Patient sociodemographic data were examined for each visit, including age, sex, race, and insurance. Age was categorized into 5 groups: 0-17 years, 18-45 years, 46-64 years, 65-74 years, and ≥75 years. Patients were grouped into 3 racial categories: White, Black, and other (which included Asian/Mideast Indian, American Indian or Alaska Native, Native Hawaiian/other Pacific Islander, more than one race, patient declined, and unknown). Insurance was categorized as Medicare (including Medicare-Medicaid Alignment Initiative), Medicaid, commercial, or other. The data were extracted from the institution’s electronic health record data warehouse. This project received a formal determination of Quality Improvement according to institutional policy. As such, this initiative was not reviewed by the Institutional Review Board.

### Statistical Analysis

First, we used descriptive statistics to examine weekly and overall visit volumes during the study period, which were the 11 weeks following the COVID-19 national emergency declaration (March 15 to May 31, 2020), compared to visit volumes in the corresponding weeks of the 2019 calendar year. Next, we examined visit type (in-person, video, telephone) and patient sociodemographics (age, sex, race, insurance) associated with the visit and compared these characteristics to those visits occurring during the same date range in 2019. Last, we examined differences in ambulatory visit type (in-person vs virtual; and for those with virtual visits, video vs telephone) by patient sociodemographics (age, sex, race, insurance) for visits occurring during the COVID-19 study period.

Data were summarized with chi-square tests where appropriate. Because of the large sample size, statistical significance was set at *P*≤.001. To estimate the association between patient sociodemographics and visit type (in-person vs virtual, and video vs phone for those with virtual visits), we performed logistic regression. Results were similar between unadjusted and adjusted analyses; only adjusted analyses are presented. Data were analyzed using RStudio, version 3.6.3 (RStudio, PBC).

## Results

### Visit Volumes and Visit Types

In the week of March 15-21, 2020, the ambulatory visit volume dropped to 34% of visit volumes when compared to the same week in 2019 (n=4877 vs n=14,343 visits) and reached a nadir of 20.8% of 2019 levels (n=2476 vs n=11,930 visits) in the following week. By the week of May 24-30, 2020, the ambulatory visit volume had rebounded to 81.8% of the volume of the same week in 2019 (n=9451 vs n=11,554 visits). Total visit volumes from March 15 to May 31, 2020, were 51.4% of 2019 volumes (n=80,081 vs n=155,884 visits).

**Figure 1:**
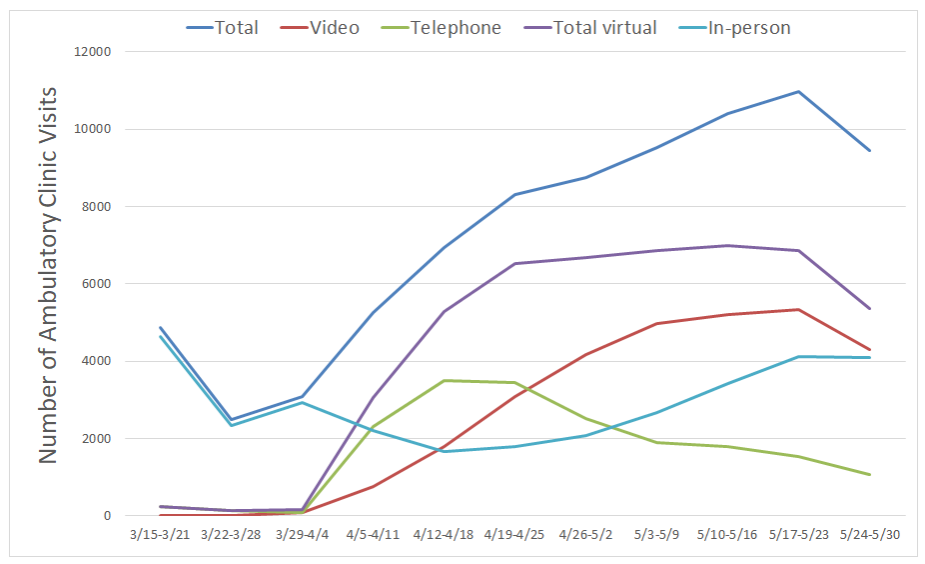
Ambulatory visit volumes and types from March 15 to May 31, 2020. Note: all visit volumes decreased during the final week of May due to Memorial Day clinic closures.

Virtual ambulatory visits increased from 0 to 48,475 visits between March 15 to May 31, 2020, and comprised 60.5% of total ambulatory visit volume, with the remaining 39.5% (n=31,606) conducted in person (Table 1 and Figure 1). Among virtual visits performed during the study period, 61.2% (n=29,661) were by video and 38.8% (n=18,814) were by telephone. For comparison, in 2019, there were no virtual visits for the same time period. Patient sociodemographics were similar for those with ambulatory visits between March 15 to May 31, 2020, and the corresponding weeks in 2019 (Table 1).

**Table 1:** Associations between patient sociodemographics and ambulatory visit type from March 15 to May 31 in 2019 and 2020.

Characteristic		Total visits in 2019 (n=155,884), n (%)	Total visits in 2020 (n=80,081)				
			Overall (n=80,081), n (%)	In-person visits (n=31,606), n (%)	Virtual visits (n=48,475), n (%)	Virtual vs in-person	
						aOR^a^ (95% CI)	*P* value^b^
**Age (years)**							<.001
	0-17	20,513 (13.2)	10,085 (12.6)	4937 (15.6)	5148 (10.6)	0.71 (0.68-0.75)	
	18-45	39,879 (25.6)	21,386 (26.7)	8192 (25.9)	13,194 (27.2)	Reference	
	46-64	43,546 (27.9)	22,283 (27.8)	8455 (26.8)	13,828 (28.5)	1.01 (0.97-1.05)	
	65-74	29,132 (18.7)	15,140 (18.9)	5957 (18.8)	9183 (19.0)	0.80 (0.76-0.84)	
	≥75	22,814 (14.6)	11,187 (14.0)	4065 (12.9)	7122 (14.7)	0.86 (0.80-0.91)	
**Sex**							<.001
	Female	95,032 (61.0)	48,571 (60.7)	18,429 (58.3)	30,142 (62.2)	Reference	
	Male	—^c^	—	—	—	0.88 (0.85-0.90)	
**Race**							<.001
	White	72,618 (46.6)	36,007 (45.0)	14,112 (44.7)	21,895 (45.2)	Reference	
	Black	65,645 (42.1)	34,852 (43.5)	14,141 (44.7)	20,711 (42.7)	0.98 (0.95-1.01)	
	Other	17,621 (11.3)	9222 (11.5)	3353 (10.6)	5869 (12.1)	1.22 (1.16-1.28)	
**Insurance**							<.001
	Commercial	53,470 (34.3)	27,642 (34.5)	9817 (31.1)	17,825 (36.8)	Reference	
	Medicare	23,663 (15.2)	11,620 (14.5)	5575 (17.6)	6045 (12.5)	1.27 (1.21-1.34)	
	Medicaid	75,100 (48.2)	39,424 (49.2)	15,169 (48.0)	24,255 (50.0)	0.74 (0.70-0.77)	
	Other	3651 (2.3)	1395 (1.8)	1045 (3.3)	350 (0.7)	0.21 (0.19-0.24)	

^a^aOR: adjusted odds ratio.

^b^Chi-square test.

^c^Not applicable.

### Association Between Ambulatory Visit Type (In-Person vs Virtual) and Patient Sociodemographics

In unadjusted analyses, there were statistically significant differences between those who received in-person and virtual visits for all sociodemographics examined (Table 1). In adjusted analyses, virtual visits were less likely than in-person visits for patients aged 0-17 years (odds ratio [OR] 0.71, 95% CI 0.68-0.75), 65-74 years (OR 0.80, 95% CI 0.76-0.84), and ≥75 years (OR 0.86, 95% CI 0.80-0.91), compared to patients aged 18-45 years (Table 1). Men were less likely (OR 0.88, 95% CI 0.85-0.90) to attend a virtual visit than women. There was no difference in the odds of virtual visit attendance between White and Black patients; however, patients with race categorized as other were more likely to attend a virtual visit (OR 1.22, 95% CI 1.16-1.28) compared to White patients. Medicare patients were more likely (OR 1.27, 95% CI 1.21-1.34) than patients with commercial insurance to attend virtual visits (vs in-person visits), whereas patients with Medicaid insurance were less likely (OR 0.74, 95% CI 0.70-0.77) than patients with commercial insurance to have virtual visits. Patients with insurance categorized as other were also less likely to have a virtual visit (OR 0.21, 95% CI 0.19-0.24) than patients with commercial insurance.

### Association Between Virtual Visit Type (Telephone vs Video) and Patient Sociodemographics for Those With Virtual Visits

In unadjusted analyses, there were statistically significant differences across all sociodemographics examined except sex between those using telephone versus video visits (Table 2). In adjusted analyses, results were similar, except there were differences by sex as well. Video visits were more likely than telephone visits for patients aged 0-17 years (OR 3.32, 95% CI 3.01-3.67), while video visits were less likely than telephone visits for patients aged 46-64 years (OR 0.56, 95% CI 0.54-0.60), 65-74 years (OR 0.47, 95% CI 0.44-0.50), and ≥75 years (OR 0.30, 95% CI 0.27-0.32), compared to patients aged 18-45 years. Men were less likely to attend a video visit (OR 0.94, 95% CI 0.90-0.97) than women. Black patients were less likely to attend a video visit (OR 0.55, 95% CI 0.52-0.57) compared to White patients. Video visits were less likely than telephone visits for Medicare patients (OR 0.69, 95% CI 0.65-0.74) and Medicaid patients (OR 0.72, 95% CI 0.67-0.77) compared to patients with commercial insurance.

**Table 2:** Associations between patient sociodemographics and type of virtual visit from March 15 to May 31, 2020.

Characteristic		Total virtual visits (n=48,475), n (%)	Virtual visits (n=48,475)			
			Telephone visits (n=18,814), n (%)	Video visits (n=29,661), n (%)	Video vs telephone	
					aOR^a^ (95% CI)	*P* value^b^
**Age (years)**						<.001
	0-17	5148 (10.6)	554 (2.9)	4594 (15.5)	3.32 (3.01-3.67)	
	18-45	13,194 (27.2)	3507 (18.6)	9687 (32.7)	Reference	
	46-64	13,828 (28.5)	5677 (30.2)	8151 (27.5)	0.56 (0.54-0.60)	
	65-74	9183 (19)	4587 (24.4)	4596 (15.5)	0.47 (0.44-0.50)	
	≥75	7122 (14.7)	4489 (23.9)	2633 (8.8)	0.30 (0.27-0.32)	
**Sex**						.17
	Female	30,142 (62.2)	11,771 (62.6)	18,371 (61.9)	Reference	
	Male	—^c^	—	—	0.94 (0.90-0.97)	
**Race**						<.001
	White	21,895 (45.2)	7084 (37.7)	14,811 (49.9)	Reference	
	Black	20,711 (42.7)	10,064 (53.4)	10,647 (35.9)	0.55 (0.52-0.57)	
	Other	5869 (12.1)	1666 (8.9)	4203 (14.2)	0.95 (0.89-1.01)	
**Insurance**						<.001
	Commercial	17,825 (36.8)	9846 (52.4)	7979 (26.9)	Reference	
	Medicare	6045 (12.5)	2127 (11.3)	3918 (13.2)	0.69 (0.65-0.74)	
	Medicaid	24,255 (50.0)	6741 (35.8)	17,514 (59.1)	0.72 (0.67-0.77)	
	Other	350 (0.7)	100 (0.5)	250 (0.8)	1.03 (0.81-1.31)	

^a^aOR: adjusted odds ratio.

^b^Chi-square test.

^c^Not applicable.

## Discussion

### Principal Findings

Total visit volumes in the COVID-19 study period were approximately half of that in 2019, although patient sociodemographics were similar. Recovery of clinic volumes after the escalation of the pandemic was largely driven by virtual ambulatory care, which comprised over 60% (n=48,475) of total ambulatory clinic volumes from March 15 through May 31, 2020, a majority of which were video visits. Children, adults ≥65 years, men, and patients with Medicaid coverage were less likely to have virtual visits, whereas patients with Medicare coverage were more likely to have virtual visits compared to patients with commercial insurance coverage. For those who attended virtual visits, children were more likely to have video visits, while adults ≥46 years, men, Black patients, and patients with Medicare or Medicaid coverage were less likely to have video visits.

The sociodemographic differences in virtual visits we identified are in line with prior research. For example, prior research found that women were more likely than men to shelter in place due to concerns about the risk of COVID-19 infection for themselves and their family; this would make virtual visits a more appealing visit type for women [25]. Additionally, studies prior to the pandemic demonstrated that women used virtual visits more often than men [11]. Similarly, patients with Medicare insurance may have been more concerned about acquiring COVID-19 infection and prefer to shelter in place, leading to their increased likelihood of attending a virtual visit. In contrast, pediatric well visits (and well visits for most non-Medicare beneficiaries) must still be performed in person to be reimbursed; therefore, many pediatric patients continued to attend in-person visits even during the COVID-19 pandemic.

The sociodemographic differences in virtual (vs in-person) visits and video (vs telephone) visits illustrate the digital divide [26]. The patient populations with lower levels of access to internet and smart devices and lower digital literacy were the same sociodemographic groups found in our study to have a lower likelihood of completing virtual or video visits, including older adults, Black patients, and patients without commercial insurance [4-9]. Our results also match prior studies on virtual visit use during the pandemic, which found that patients using virtual visits during the pandemic were more likely to be younger adults as compared to older adults, White, and commercially insured [21-23]. Requirements for a video visit include internet, a capable device, and a basic level of digital literacy, so patients who do not have all three (or do not have a readily available family member to assist) are unable to attend video visits. One study performed during the pandemic found higher prevalence of “unreadiness” to attend video visits in those sociodemographic groups found to be less likely to attend video visits, including patients who were older, Black, and men, similar to our findings [24]. These findings raise concerns about the role video visits may play in exacerbating existing health inequities, particularly since the quality of health care visits and visit satisfaction are greater with video visits compared to telephone visits [11-13]. Moreover, these health disparities may be significantly worsened if the current reimbursement parity between telephone and video visits is discontinued, and especially if telephone visits are no longer reimbursed altogether following the public health emergency.

The shift in the delivery of ambulatory care through virtual visits was incentivized by the new virtual reimbursement policies from CMS and private insurance companies. The significant contribution of virtual visits to overall ambulatory visit volumes is likely to continue once the COVID-19 pandemic has ended. The volume of virtual ambulatory visits at UCMC has continued to grow even after the end of the study period, indicating sustained interest in virtual visits likely due to continued safety concerns related to the pandemic, ongoing reimbursement for these services, and physician and patient satisfaction with this new option for care delivery [27,28]. Given the interest in and development of virtual visits prior to the pandemic and the proliferation of virtual visits during the pandemic, virtual visits for ambulatory care are likely to remain popular among both patients and providers even after the COVID-19 pandemic [1,2]. University of Chicago Medicine’s 2025 Strategic Vision (developed prior to the pandemic) includes an “aim to build a digitally enabled organization for patients” and a goal to expand access to care, both of which are aided by the expansion of virtual visit services [29]. However, if reimbursement for virtual visits is discontinued or significantly reduced after the pandemic or public health emergency ends, many medical centers are likely to stop making significant investments in the continued development of their telemedicine programs and the availability of virtual visits for patients would be expected to decline.

### Recommendations

The results of this study and our review of the virtual visit landscape has prompted us to offer five recommendations (Textbox 1). First, given the differences in virtual visit use by certain sociodemographic groups demonstrated in this study and the lower effective reimbursement rates for telephone visits compared to video visits, medical institutions like UCMC with high proportions of older, Black, and/or Medicare/Medicaid patients may experience lower reimbursement rates because of the barriers these groups face to completing video visits. For a video visit, providers can bill for all time spent on patient care on the encounter date, including documentation; for a telephone visit, they can only bill for time spent in direct communication (on the telephone call) with a patient on the encounter date. To avoid effectively penalizing medical institutions providing care to vulnerable populations, government and commercial insurers should help address these disparities by *maintaining reimbursement parity between video and telephone visits*. Second, given the rapid growth and early success of virtual visits, and the role they will likely play in blended models of care, *legislation that makes virtual visit reimbursement permanent* is essential to allow for the long-term investment by health care systems and providers needed to improve the virtual visit infrastructure and experience. Third, government insurers and specialty societies should collaborate to *establish guidance to help distinguish ambulatory care best suited for virtual versus in-person care*. Fourth, quality improvement initiatives should be undertaken at medical institutions to *support and improve access to and usability of video visits* in populations encountering the greatest barriers to its use. Last, *advocacy for policy changes and more universal broadband access* are essential to help close the digital divide experienced by our most vulnerable patient populations, which would help address the differential access to virtual visits described in this study.

**Recommendations to improve access to and use of virtual visits.**

1. Maintain reimbursement parity between video and telephone visits
2. Pass legislation making virtual visit reimbursement permanent
3. Establish guidance to distinguish ambulatory care best suited for virtual versus in-person care
4. Perform quality improvement initiatives to improve access to and usability of video visits in vulnerable populations
5. Advocate for policy changes and universal broadband access to close the digital divide

### Limitations

Our study has limitations. First, this study only examined a single medical center and was a retrospective analysis; despite this, the diversity of the patient population examined in our study enabled our analysis of ambulatory virtual visit use. Second, our study only examined a limited set of variables, which were used as surrogates for the social determinants of health described in this paper, such as access to broadband internet, health literacy, tech literacy, education, and income, and did not examine virtual and video visit use by ethnicity due to limited data availability. Third, this area of clinical practice and study is rapidly changing and will likely continue to change rapidly over the next few months to years. Further studies at other medical institutions should be conducted to confirm our findings and examine additional sociodemographic variables. Future analyses of ambulatory virtual visits should also investigate patient satisfaction and outcomes by patient visit type (eg, new, return, consult), given the differences in reimbursement by visit type category, and whether ambulatory virtual visits increase the geographic area served by academic medical centers or medical institutions with subspecialty care, as already suggested by limited data [30].

### Conclusion

The COVID-19 pandemic has drastically changed the health care delivery landscape largely due to the growth of ambulatory virtual visits, which have rapidly become a vital component of health care delivery. Given the differential use of these technologies by age, sex, race, and insurance, these changes also risk perpetuating and even exacerbating existing disparities in health care access and quality, especially if reimbursement policies do not sufficiently account for these differences and the digital divide remains unaddressed.

## Abbreviations

CMS: Centers for Medicare & Medicaid Services
HIPAA: Health Insurance Portability and Accountability Act
OR: odds ratio
UCMC: University of Chicago Medical Center
